# Racial Imposter Syndrome and Music Performance Anxiety: A Case Study

**DOI:** 10.3390/bs15081057

**Published:** 2025-08-04

**Authors:** Trisnasari Fraser

**Affiliations:** Independent Researcher, Melbourne 3085, Australia; info@iamreadypsychology.com

**Keywords:** music performance anxiety, racial imposter syndrome, acceptance and commitment therapy, attachment-based therapy

## Abstract

The impact of cultural identity on music performance anxiety (MPA) is under-researched. This retrospective case study explores the treatment of a professional musician in her 30s who presented with MPA associated with performing music related to her estranged father’s cultural background. The case formulation identified attachment ruptures and negative cognitions associated with her mixed cultural heritage that contributed to an experience of imposterism—referred to in lay literature as ‘racial imposter syndrome’ (RIS). It was hypothesized that RIS served to perpetuate her MPA. An attachment-based approach and Acceptance and Commitment Therapy framework was adopted, drawing on evidence-based treatment for MPA and mixed heritage individuals. The Depression Anxiety Stress Scale-21 (DASS-21), Outcome Rating Scale (ORS) and Session Rating Scale (SRS) were used as outcome measures. These measures fluctuated throughout the therapy. While improvements were observed in depression scores midway through treatment, elevated stress and depression scores at the conclusion of treatment were understood to reflect situational factors related to financial and housing precarity. Nonetheless, at the conclusion of treatment, the client showed improvement in managing MPA, evidenced by her progress in recording an album and reengagement with public performances. This case study adds to the limited research on treating MPA in racially minoritized and mixed-race individuals, Further research is required across larger and more diverse samples to better understand the relationship between MPA and RIS and to develop effective interventions.

## 1. Introduction

Music performance anxiety (MPA) is understood to be associated with fears of negative social evaluation (Kenny, 2011; Guyon et al., 2022; Nicholl & Abbott, 2024) and is classified in the Diagnostic and Statistical Manual of Mental Disorders (5th ed.; DSM-5; American Psychiatric Association, 2013) as a specifier within social anxiety. Imposter phenomenon (IP), or imposter syndrome in the lay literature (Bravata et al., 2020), while not a diagnostic category in the DSM-5, is an internal experience of persistent self-doubt about one’s performance (Clance & Imes, 1978; Gadsby & Hohwy, 2024). Like MPA, IP has also been associated with negative social evaluation (Mak et al., 2019; Yaffe, 2021). Music performance anxiety and IP also share associations with high levels of perfectionism, shame, low self-esteem, and procrastination (Boyett, 2019; Bravata et al., 2020; Chrousos et al., 2020; Coşkun-Şentürk & Çırakoğlu, 2018). Negative mindsets and scrutiny by self or others have been found to be factors with the highest correlation across the two constructs (Sims & Ryan, 2023).

Imposter phenomenon has been found to be prevalent among ethnic minorities (Bravata et al., 2020; Nadal et al., 2021), and in more recent years, the concept of racial imposter syndrome (RIS) has gained attention, mainly outside academic and clinical literature (Donella, 2018; Lacey, 2022; Salhany, 2022). Racial imposter syndrome describes the experience of some people of mixed heritage, typified by identity confusion (Donella, 2018), and feelings of self-doubt associated with a misalliance between how others perceive them and their own sense of their racial or ethnic identity (Lacey, 2022; Salhany, 2022).

Imposter phenomenon is thus known in clinical literature as it characterizes a widely experienced phenomenon, rather than a formal diagnosis; however, a case has been made for the inclusion of IP in the DSM-5 to codify treatment (Bravata et al., 2020). Imposter phenomenon is often found to be co-morbid with depression and anxiety; accordingly, Bravata et al. (2020) argued that health professionals are likely to treat clients presenting with IP for these co-morbid conditions. The recent rise in peer-reviewed literature about IP is attributed to the proliferation of lay literature related to imposter syndrome (Bravata et al., 2020). While there has been interest in RIS in lay literature, this has not been reflected in peer-reviewed literature; however, there has been discussion about the need for a reconceptualization of a racialized IP (Cokley et al., 2024). Given the scant peer-reviewed literature, theorization and research related to IP and the mental health outcomes of individuals of mixed heritage is drawn upon to develop theory about the aetiology of RIS in the following overview.

### 1.1. Aetiology of Music Performance Anxiety and Racial Imposter Syndrome

The degrees to which MPA and IP are influenced by nature or nurture have been considered in the literature and are elaborated further below. Theorization of MPA has drawn on Barlow’s (2000) model, which proposes three vulnerabilities to the development of an anxiety disorder, including (1) biological—heritability; (2) generalized psychological—based on early experience of developing a sense of control over salient events; and (3) specific psychological—where anxiety becomes associated with a specific object or situation through learning processes (Kenny et al., 2004; Kirsner et al., 2023; Osborne & Kenny, 2008). Consistent with this theorization, high trait anxiety and negative cognitions related to aversive performance incidents have been found to be predictive of MPA (Osborne & Kenny, 2008). Likewise, MPA and early maladaptive schemas—pervasive themes or patterns dysfunctionally characterizing an individual’s relationship with others—have been found to be highly correlated and associated with recollections of critical and overprotective parenting (Kirsner et al., 2023). Finally, theorization of MPA has considered the appraisal of emotional and physiological demands arising from performance, with the reappraisal of anxiety as excitement understood to facilitate performance (Brooks, 2014; Nicholl & Abbott, 2024; Osborne et al., 2020; Twitchell et al., 2025).

In a study of correlations between MPA and IP in graduate music performance students, Sims and Ryan (2023) found correlations for factors related to biological vulnerability and parental empathy had a weaker relationship with IP, suggesting individual attributes such as temperament and training experiences may be more strongly associated with development of IP. However, initial theorization of IP by Clance and Imes (1978) emphasized the role of early family dynamics. Negative or idealized narratives about intellectual ability internalized in early development were understood to lead to differentials between the expectations and experiences of intellectual achievement among the highly successful, primarily white women in their study. Imposter phenomenon has since been considered in other populations. The argument for reconceptualization of a racialized IP stems from the environmental circumstances particular to racially and ethnically minoritized individuals (Cokley et al., 2024). Cokley and colleagues (2024) questioned the use of scales that measure IP as a stable trait for racially and ethnically minoritized individuals, for whom IP may be better characterized as a normal response to discriminatory environments.

Integrating literature about microaggressions, stereotype threat and IP, as well as social identity and ecological theory, Nadal et al. (2021) developed a model to describe how cumulative effects of microaggressions contribute to internalized oppression—negative beliefs about one’s identity groups. Drawing on ecological theory (Bronfenbrenner, 1992), Nadal et al. (2021) theorized that the experience of microaggressions and overt discrimination from society, family members and peers can accumulate, with negative implications for self-esteem, IP and mental health. High racial centrality—the degree to which race is an important aspect of identity—has been found to be a protective factor against IP (Nadal et al., 2021), and thus, the authors theorized that consistent with social identity theory (Tajfel et al., 1979), positive racial socialization can boost resilience against these effects. Furthermore, Nadal et al. (2021) cited literature that found people of colour who externalize racism, by holding systems accountable rather than themselves, were likely to live longer.

The implications for RIS are complex. For individuals of mixed heritage, high racial centrality may be difficult to foster, and this may be influenced by exclusionary messages received from society, family members and peers. Anecdotally, the experience of not being recognized as belonging, by others in one’s identity group, contributes to RIS (Donella, 2018; Lacey, 2022; Salhany, 2022). Furthermore, being “white-passing” can contribute to confusing cognitions connected to identity as it relates to the systemic underpinnings of racism. Lacey (2022) provided an exemplar of such cognitions, reflecting on her role as an educator in information literacy, and identity as a woman of Sri Lankan and English ancestry. She articulated her dilemma as follows:

After eight years of teaching, I still get nervous being in front of a class. More specifically, I assume that students see me as a white woman, that this is the identity they place on me. As a result, I have been unsure of how to present myself in these spaces, whether it is okay to identify as not-white while experiencing white privilege, if I am taking up too much space outlining my identity in the one-shot classroom, and whether it benefits anyone but myself to make that identity clear.

(Lacey, 2022, p. 842)

Indeed, people of mixed heritage have been found to have poorer mental health outcomes compared to those who identify with only one racial or ethnic group (Oh et al., 2024). Families that do not look alike due to mixed heritage have been observed to be scrutinized, with white family members experiencing minoritization in certain social contexts (Klocker & Mbenna, 2025).

### 1.2. Treatment of Music Performance Anxiety and Racial Imposter Syndrome

In the absence of treatment protocols for MPA and RIS, the following section reviews viable treatment options, based on the aetiologies theorized above and available empirical literature regarding interventions. The link between emotional dysregulation and relationships in early development may be relevant for both MPA and RIS. For MPA, maladaptive schemas may emerge from the experience of rupture with significant caregivers. For RIS, they may emerge from the experience of racial microaggressions from family, peers or the wider society. Attachment-based therapeutic approaches, which seek to resolve attachment ruptures through the therapeutic process, have been used in the treatment of MPA (Kenny & Holmes, 2015; Kenny, 2016). Likewise, they have been used in the treatment of racial trauma for diasporic communities and mixed heritage individuals who experience “the absence of a secure base nation or place of belonging” (Davis, 2007). There is insufficient data available to draw firm conclusions about the effectiveness of these approaches for such presentations; however, they are reported favourably via case reports (Davis, 2007; Kenny & Holmes, 2015; Kenny, 2016) and thus warrant further investigation.

Attachment theory considers how the emotional bonds developed with significant caregivers early in life can have enduring influence on relationships (Bowlby, 1969; Ainsworth, 1989). Expanding on three attachment styles proposed by Ainsworth (1989), Bartholomew and Horowitz (1991) proposed four attachment styles including secure, preoccupied, fearful-avoidant, and dismissive-avoidant. A secure attachment style is associated with a positive self-regard and the ability to be either self-reliant or seek help when needed. An anxious-preoccupied attachment style is associated with a sense of unworthiness and high regard for others and is characterized by a desire for closeness together with a fear of rejection. A fearful-avoidant attachment style is associated with a sense of unworthiness and the expectation that others will be negatively disposed and is characterized by avoidance of close involvement with others. Finally, a dismissive-avoidant attachment style is associated with feeling worthy of love but negatively disposed toward others and is characterized by detached or dismissive attitudes toward close relationships. Attachment-based therapy uses attachment theory to both understand a client’s experiences and create change based on the attachment relationship between therapist and client (Slade & Holmes, 2019). The use of attachment theory in culturally diverse populations requires an understanding of culture specific conceptualizations of early care-giving (D. Brown et al., 2008), and consideration of how racialized experience influences attachment behaviour (Stern et al., 2021).

Acceptance and commitment therapy (ACT) has likewise been used in the treatment of both MPA (Clarke et al., 2020; Juncos & de Paiva e Pona, 2018; Juncos et al., 2017; Juncos & Markman, 2016), and racially minoritized populations (Misra et al., 2023). Like attachment-based therapy, the available data on the effectiveness of ACT for MPA and racially minoritized populations is very promising; however, small sample sizes necessitate caution in drawing strong conclusions. While evidence for the efficacy of ACT as a treatment for social anxiety and anxiety disorders over traditional Cognitive Behavioural Therapy (tCBT) has been cited in the literature about the use of ACT for treatment of MPA (Clarke et al., 2020; Juncos & de Paiva e Pona, 2018), recent comparisons between the effectiveness of tCBT and ACT are equivocal (Fang & Ding, 2023). Fang and Ding (2023) conducted a meta-analysis comparing ACT and tCBT and found the latter to be more effective at anxiety symptom reduction and the former to be more effective in improving psychological flexibility. The rationale for using ACT over tCBT is supported by the advantage conferred to performers by the release of stress hormones, with appropriate appraisal of the situation as challenge rather than threat (Brooks, 2014; Nicholl & Abbott, 2024; Osborne et al., 2020; Twitchell et al., 2025). Relatedly, Juncos and de Paiva e Pona (2018) noted that musicians experiencing MPA may not be seeking anxiety symptom reduction as much as an improvement in performance skills. While both tCBT and ACT use approaches such as exposure, ACT diverges from tCBT in that the goal is not a reduction in symptoms but an increase in psychological flexibility for the client to continue to move toward values and goals regardless of immediate thoughts and feelings (Levin et al., 2017).

Strategies to “unhook” from thoughts and feelings to pursue value-based action are informed by the ACT hexaflex, comprising six interrelated components, including (i) contact with the present moment; (ii) defusion; (iii) acceptance; (iv) self as context; (v) values; and (vi) committed action (Harris, 2009). Non-judgemental contact with the present moment, defusion and acceptance are strategies to mitigate experiential avoidance—attempts to change or control unwanted thoughts, feelings or physiological sensations. Self as context encourages the client to observe their ever-changing experience with curiosity and not as the essence of who they are. Clients are supported in clarifying their underlying values—how they derive meaning from life. Values are used as a compass to underpin committed action in service of these values, regardless of the vicissitudes of life experience.

Kinney et al. (2025), in this edition, noted that a large proportion of MPA interventions are multimodal, often comprising aspects of CBT, relaxation techniques, goal setting and other psychological skills training. The authors offered two conjectures about this trend—either that an integrated approach was required to address the complex aetiology of MPA, or that the emergent nature of the field gave rise to more experimental approaches.

### 1.3. The Present Study

This case concerns “Mary”, a professional musician in her 30s, referred to private psychological treatment by her general practitioner (GP), who cited functional impairment, RIS, internalized racism and interpersonal concerns as presenting issues. In the assessment sessions, Mary indicated that interpersonal difficulties were associated with anticipatory anxiety regarding upcoming music performances and recordings. While important details of the current case are accurate, identifying features of Mary have been altered to maintain anonymity.

## 2. Materials and Methods

### 2.1. Informed Consent

Informed consent for therapy was obtained from the client in writing at the beginning of treatment and revisited throughout the therapy via ongoing communication. Following conclusion of therapy, written informed consent was obtained from the client to publish this paper, in accordance with COPE guidelines (Barbour, 2016). As a retrospective review by a private practitioner, institutional review board (IRB) approval was not sought for this case study. In Australia, many IRBs connected to health institutions consider case reports or studies to be anecdotal and exempt from ethics review (e.g., EMHS, 2020; RCH, n.d.).

### 2.2. Case Formulation

The following case formulation was developed from GP referral, client report, clinician observation and quantitative measure (as outlined below). Mary presented as articulate, with good insight and keen to engage in therapy. She stated her goals were to address self-sabotage, procrastination, and feelings of imposterism, renew focus, purpose, and intentionality in alignment with personal values, and attend to interpersonal difficulties and negative thinking patterns. When providing an account of interpersonal conflict, she demonstrated marked distress and described how the conflict revolved around prior and upcoming music performances. She indicated that discussion about exotification and cultural appropriation had intensified among a group of musicians with whom she was collaborating. The discussions had caused a rupture and precipitated Mary’s experience of RIS. While Mary had future performances and recordings with some of the musicians, consistent with MPA, she reported approaching these with a sense of dread and significant procrastination. A formal inventory to measure MPA was not used due to client identified priorities.

While Mary was of mixed heritage, she was raised by her mother who was Anglo-Australian, following family estrangement from her father prior to her reaching school-age. She described feeling “othered” by her Anglo-Australian extended family due to her appearance; however, she also expressed feeling like an imposter when identifying with her father’s cultural background. This latter observation was corroborated by her GP’s referral which identified RIS and internalized racism as presenting issues. In recent years, she had explored music traditions of her father’s culture and integrated them into her music practice. The experience of feeling othered early in development was hypothesized to contribute to ongoing fears of abandonment, which was consistent with the significant distress displayed when recounting interpersonal ruptures. Based on these observations, it was hypothesized that Mary’s primary attachment style was anxious-preoccupied. This attachment style, and associated negative cognitions, together with perceived scrutiny about her practicing the music traditions of her father’s culture in the Australian social and cultural context were understood to perpetuate her MPA.

Like many musicians, Mary managed a portfolio career comprising freelance work, including performance, conducting workshops, and contract administrative work (Bartleet et al., 2020). As such, stressors related to financial precarity and housing were also considered to be perpetuating factors, as part of her income was tied to a practice that was causing distress. Among protective factors for Mary were her good insight, willingness to engage in therapy, a regular yoga practice and social support via housemates and friends from high school. In the results and discussion, consideration of Mary’s engagement with her father’s culture as both a factor precipitating her RIS and as a protective factor will be expanded upon.

### 2.3. Materials

Three self-report measures were administered at intervals through the therapy, including the Depression Anxiety Stress Scale-21 (DASS-21), Outcome Rating Scale (ORS) and the Session Rating Scale (SRS).

#### 2.3.1. Depression Anxiety Stress Scale-21

The DASS-21 includes 7 items from each of three subscales measuring symptoms of depression, anxiety and stress (Lovibond & Lovibond, 1995). A review of the measurement properties of the scale across 48 studies demonstrated high-quality evidence for bi-factor structural validity, internal consistency and hypothesis testing of construct validity (Lee et al., 2019).

The DASS-21 was administered in the first session. Stress and anxiety were in the normal range. Depression was in the mild range. This is consistent with client report in the initial clinical interview, where Mary indicated that she did not experience marked physiological symptoms of anxiety, but rather a lack of motivation to approach the tasks of recording and preparing for performance. It was hypothesized this may also reflect experiential avoidance, where Mary procrastinated on the tasks primarily to avoid the cognitive dissonance and associated stress of engaging in a practice that brought fears of not belonging to the fore and had been perceived to have attracted scrutiny.

#### 2.3.2. Outcome Rating Scale

The ORS (Miller et al., 2003) was developed as a brief outcome evaluation measure, to balance the need for routine outcome evaluation in therapy with feasibility of regular administration (Bringhurst et al., 2006). The ORS measures four dimensions of client functioning including personal well-being; interpersonal well-being; social role; and overall well-being, along four visual analogue scales which are l0cm lines. A review of 24 studies analyzing the reliability and validity of the ORS with clinical populations showed moderately high to high internal consistency (Harris et al., 2019). Harris and colleagues noted the ORS may be best thought of as a measure of quality of life and a potential screening tool for depressive symptoms, based on concurrent validity results. During treatment, the ORS was used to prompt further investigation of current stressors and in turn refine the case formulation and treatment plan. In the first session, Mary scored six (out of ten) on all four dimensions. Both the DASS-21 and ORS results suggested a subclinical presentation.

#### 2.3.3. Session Rating Scale

The SRS was designed as a measure of the working alliance between client and therapist (Duncan et al., 2003). Duncan and colleagues noted that the brevity of the SRS was a response to resistance to the use of longer alliance-based measures by therapists. Like the ORS, the SRS uses 10 cm long visual analogue scales to measure four dimensions of the working alliance, including the relational bond between the therapist and client, agreement on the goals of therapy, agreement on the approach or method of therapy and the overall confidence the client has that therapy and the therapist will be helpful (Duncan et al., 2003). A review of 12 studies showed high internal consistency for the SRS and test–retest reliability measures ranging from 0.54 to 0.70 (Murphy et al., 2020). Over the course of the therapy, the SRS was administered both to measure working alliance and facilitate exploration of attachment within the therapeutic context. To this end, it formed a part of the attachment-based therapy approach, which is expanded upon in the procedure section below. In the first session, Mary scored eight (out of ten) on all four dimensions.

### 2.4. Procedure

Mary attended six treatment sessions in 2023 and six treatment sessions in 2024 at intervals of between a fortnight and one month. Sessions were one hour in duration. Sessions 1 and 2 comprised initial assessment and development of the case formulation.

#### 2.4.1. Attachment-Based Therapy

As noted in the case formulation, consistent with an anxious-preoccupied attachment style, Mary displayed significant distress when discussing interpersonal ruptures. Accordingly, containment or deactivating strategies were employed to help Mary manage her distress (Slade & Holmes, 2019). These included the use of breathing and relaxation techniques in session to help Mary downregulate and access her window of tolerance—an optimal zone of arousal for functioning (Siegel, 1999). Within this zone, Mary was better able to use a range of metacognitive skills to explore and come to an understanding of patterns of response and maladaptive schemas (D. P. Brown & Elliott, 2016). This was facilitated using tCBT approaches of Socratic questioning and downward arrow technique, where the therapist encourages self-questioning to drill down to underlying beliefs (Burns, 1999).

Mary was also encouraged to consider the process over the content of the conflict, to apply metacognitive skills to the rupture—considering other people’s communication styles and emotional responses as well as her own. While much of this work occurred in sessions 3 to 5, these techniques were used throughout the treatment.

In addition to fostering a range of metacognitive skills, D. P. Brown and Elliott (2016) outlined two other pillars of attachment-based therapy including (1) the therapist modelling an ideal parent figure by listening compassionately and attending to needs that may have been unmet in early development; and (2) fostering collaborative behaviour in treatment. In Mary’s treatment, these pillars were pursued via the general attitude of the therapist, client-centred approaches and collaborative goal setting, as well as open communication about interpersonal processes occurring in the therapeutic relationship. As noted above, this was sometimes facilitated using the SRS. Session 6 comprised a review of the therapeutic goals and interventions, which also served to strengthen the therapeutic alliance.

#### 2.4.2. Acceptance and Commitment Therapy

Fostering metacognitive skills aligned with the “self as context” element of the ACT hexaflex, encouraging Mary to non-judgementally observe her thoughts, feelings and actions both in relation to interpersonal conflict and her anticipation of performing and recording. Mindfulness techniques likewise aligned with stress management strategies employed to downregulate Mary in times of significant distress. These techniques, including guided meditation and grounding, were used to encourage contact with the present moment and acceptance of the range of arising thoughts and feelings. In collaboration with the therapist, Mary completed a series of worksheets providing psychoeducation about defusion techniques and facilitating clarification of values and goals (Harris, 2010; Harris, 2016). The use of metaphor was used to help Mary distance or defuse from maladaptive schemas and negative thinking patterns (Saulsman, 2025). Introduction to ACT principles commenced in session 4 and continued throughout the treatment, with progress toward goals revisited regularly, with defusion and mindfulness techniques reinforced to help overcome obstacles.

## 3. Results

**Figure 1:**
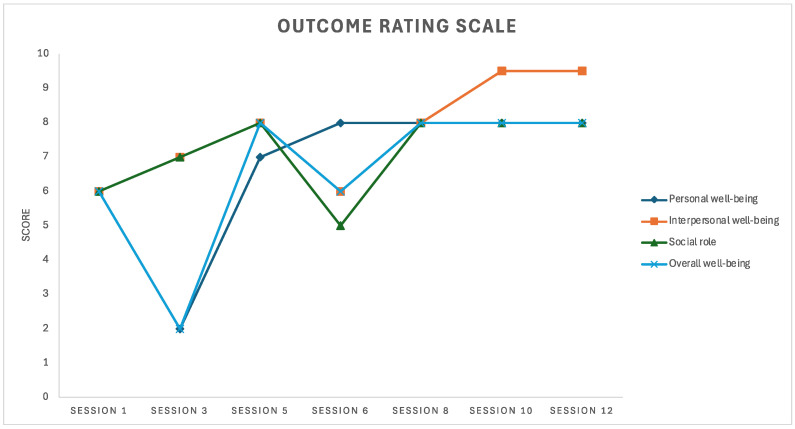
Outcome Rating Scale scores over the course of therapy.

**Figure 2:**
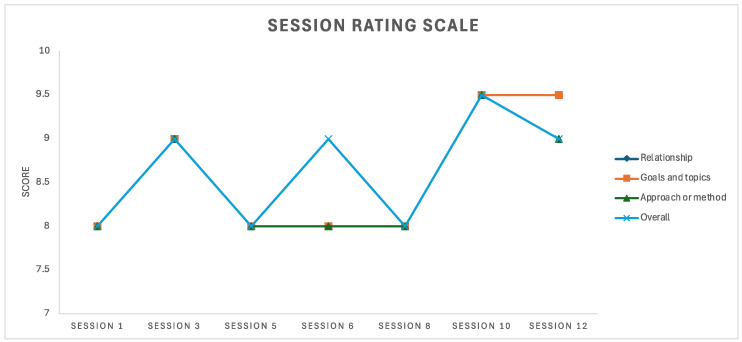
Session Rating Scale scores over the course of therapy.

The DASS-21 was administered again in the sixth and twelfth sessions. In the sixth session, stress, anxiety and depression were in the normal range. In the twelfth session, anxiety was in the normal range, and stress and depression were in the mild range. The elevated stress and depression scores in the twelfth session were understood to relate to situational factors related to financial and housing precarity. The ORS and SRS were administered seven times over the twelve sessions. The results are presented in Figure 1 and Figure 2 and illustrate some fluctuation in functioning via the ORS (Figure 1) and small fluctuations in a generally strong working alliance via the SRS (Figure 2).

During therapy, Mary established contact with extended family and arranged a month-long stay in her father’s country of origin to visit and engage in an immersive language learning program. This was understood to align with a personal goal identified by Mary to work toward integrating cultural identity and spirituality. While her initial engagement with the music traditions of her father’s culture had precipitated RIS and by association MPA, further engagement with the culture was understood to serve as a protective factor. It had contributed to Mary better integrating her cultural identity and strengthening racial centrality. Mary made progress with the recording of an album and reengaged with public performances drawing on the musical traditions of her father’s culture. Mary also made progress with a goal of establishing a more stable financial status by investigating teacher training. While interpersonal conflicts continued to arise from time to time, Mary appeared less distressed when recounting these in session and demonstrated an ability to apply metacognitive skills to contemplate and problem solve these disagreements.

## 4. Discussion

The present case study contributes to the limited literature exploring the treatment of MPA among racially minoritized populations and individuals of mixed-race heritage. The findings highlight the potential effectiveness of an integrative approach combining attachment-based therapy and ACT in addressing complex presentations of this nature.

The case of Mary illustrates an interplay between MPA and RIS, where her anticipatory anxiety about music performance was exacerbated by perceived social scrutiny about her cultural identity and the appropriateness of her performing these traditions within the Australian social and cultural context. This aligns with the theorized aetiology of MPA, which considers biological vulnerabilities, early experiences shaping psychological vulnerabilities, and specific learning processes associating anxiety with performance situations (Barlow, 2000; Kenny et al., 2004; Osborne & Kenny, 2008). Additionally, the case highlights the role of socio-cultural factors, such as racial microaggressions in contributing to the development of IP (Nadal et al., 2021).

The attachment-based approach adopted in this case aimed to resolve attachment ruptures and maladaptive schemas stemming from Mary’s early experiences of feeling “othered” within her family. By fostering a secure therapeutic relationship and employing strategies like containment, metacognitive skills development, and collaborative goal-setting, the therapist facilitated Mary’s exploration of interpersonal patterns and underlying beliefs (D. P. Brown & Elliott, 2016; Slade & Holmes, 2019). This approach aligns with the theorized role of early parental experiences and attachment styles in the development of MPA (Kirsner et al., 2023) and the potential for attachment-based therapy to address the absence of a secure base for individuals of mixed heritage (Davis, 2007).

Concurrently, the ACT framework provided strategies to increase psychological flexibility and promote value-driven action, despite the presence of distressing thoughts and feelings associated with MPA and RIS. Techniques such as mindfulness, defusion, and values clarification enabled Mary to disentangle from negative cognitions, accept her internal experiences, and engage in committed action aligned with her personal values (Harris, 2009). This approach resonates with the rationale for using ACT in MPA treatment, where the goal is not symptom reduction but improved performance readiness and psychological flexibility (Juncos & de Paiva e Pona, 2018; Osborne et al., 2020).

The outcome measures, including the DASS-21, ORS, and SRS, demonstrated fluctuations in Mary’s functioning and the therapeutic alliance throughout the treatment process. The elevated DASS-21 stress and depression scores in the final session highlights the potential impact of situational stressors, such as financial and housing precarity, on Mary’s well-being. This is of relevance to many professional musicians and underscores the importance of considering broader social factors that may contribute to the maintenance of MPA. Music performance can be experienced as high pressure both due to social evaluation and being linked to financial stability. While this case study contributes to an understanding of MPA among mixed heritage individuals, financial precarity is a factor relevant to a large proportion of professional musicians and thus the case emphasizes the importance of considering social context in formulation and treatment of MPA more broadly.

Notably, Mary’s engagement with her cultural heritage can be viewed as both a precipitating factor for her presentations and a protective factor. While initially contributing to her distress and interpersonal conflicts, this engagement ultimately facilitated Mary’s integration of her cultural identity and spirituality, aligning with her personal values. This outcome resonates with the theorized protective role of positive racial socialization and high racial centrality in mitigating the effects of internalized oppression and microaggressions (Nadal et al., 2021).

Limitations of the present study include the absence of formal measurement of MPA, limiting the precision with which treatment outcomes can be assessed, and the lack of follow-up data to assess the maintenance of treatment gains. Additionally, the case study focuses on a single client, limiting the generalizability of the findings. Further research is required to improve understanding of RIS as a phenomenon, and to investigate the intersection between MPA and RIS. Future studies should aim to employ mixed-methods approaches and formal MPA assessment tools (e.g., K-MPAI, Kenny, 2009), combined with measures that assess racial identity development, experiences of microaggressions, and internalized oppression. Longitudinal and qualitative designs could shed light on how RIS and MPA evolve over time and how they interact within different sociocultural contexts.

In the context of higher music education, such research could inform the development of culturally responsive pedagogical practices. Understanding how identity-based anxieties affect musical performance and well-being may encourage institutions to move beyond generic performance coaching and mental skills training toward more holistic, identity-informed interventions. This has implications not only for improving student outcomes but also for fostering inclusive learning environments that recognize and address the unique stressors faced by racially minoritized and mixed-heritage students.

More broadly, exploring MPA through the lens of RIS and other socio-cultural stressors can deepen our understanding of MPA as a socially situated phenomenon, rather than a purely individual or performance-specific issue. Future research in this area could contribute to more nuanced, contextualized models of MPA and guide the development of interventions that incorporate cultural, relational, and systemic dimensions.

## Data Availability

Data is unavailable due to privacy and ethical reasons.
